# An association of spleen volume and aortic diameter in patients and in mice with abdominal aortic aneurysm

**DOI:** 10.1186/s12893-017-0328-5

**Published:** 2017-12-15

**Authors:** Fang-Da Li, Rui Kang, Hao Nie, Xi-Ming Wang, Yue-Hong Zheng

**Affiliations:** 1 Department of Vascular Surgery, Peking Union Medical College Hospital, No.1 Shuaifuyuan, Dongdan, Dongcheng District, Beijing, 100730 China; 2 Shandong Medical Imaging Research Institute, Shandong Provincial Key Laboratory of Diagnosis and Treatment of Cardio-Cerebral Vascular Diseases, Shandong University, NO, 324, Jingwu Road, Jinan, Shandong 250021 China

**Keywords:** Abdominal aortic aneurysm, Inflammation, Ruptured, Imaging, Spleen volume

## Abstract

**Background:**

To investigate the potential mechanism of splenic enlargement in Ang II/APOE model and the associations between the spleen volume and the indices of abdominal aortic aneurysm (AAA) in human.

**Methods:**

To investigate the changes of spleen volume on AAA formation, apolipoprotein E knockout (Apo E^−/−^) mice were treated with Ang II (1000 ng/kg/min) up to 28 days to generate AAA. We used Magnetic Resonance Imaging (MRI), liquid measurement, H&E and immunohistochemistry to analyze the morphological or pathological changes of spleen. To investigate the changes of spleen volume in human, a retrospective case-control study involving 30 male AAA patients and 25 male controls were performed. Spleen volume was measured on computed tomography images. Univariate analysis and multivariable sequential logistic regression analyses were used to analyze the association between spleen volume and maximal diameter (Dmax).

**Results:**

In Ang II/APOE model, we found splenic enlargement in mice with AAA compared with the sham group. Histopathological investigations revealed hypertrophies of splenic follicles and increased populations of CD3^+^ T cells. In clinic cohort study, univariate analysis revealed higher values in large AAA (Dmax > 5.5 cm,*n* = 15) compared with the small (Dmax < 5.5 cm,n = 15) for spleen volume (230.6 ± 64.5 cm^3^ vs. 170.0 ± 32.8 cm^3^; *P* = 0.0030). Regression analysis revealed a statistically significant positive linear correlation of spleen volume and Dmax of AAA (*r* = 0.3611;*P* = 0.0423).

**Conclusions:**

Mimicking the splenic pathology observed in murine AAA model, there is a strong positive correlation between spleen volume and the Dmax in male AAA patients. As Dmax is a valuable predictor of AAA rupture, the spleen enlargement may be another indicator.

## Background

Abdominal aortic aneurysm (AAA) is a common vascular disease of the abdominal aorta, characterized by degeneration of the medial wall and various degrees of chronic aortic wall inflammation [1]. According to contemporary population-based data from Northern Europe, AAAs are found in about 2.2% of 65-year-old men [2]. Ruptured AAA is one of the leading causes of death among elderly men [1]. The main clinical indicators used to assess the risk of rupture are the maximum abdominal aortic diameter (Dmax) and expansion rate of the AAA obtained from ultrasound or computed tomography angiography (CTA) scanning. Surgery is recommended when the Dmax of AAA measures > 5.5 cm or when Dmax expands 1 cm/y for smaller AAAs [3]. Small aneurysms can also rupture, however, and the overall mortality rate associated with these may exceed 50% [4]. Therefore, more reliable criteria associated with the actual rupture potential of the AAAs are needed to improve patient selection for surgery or endovascular stenting.

**Table 1:** Background characteristics of the patients

	Control	AAA	*P* value
Number of cases	25	30	
Age,years	55.7 ± 4.8	67.7 ± 7.5	<0.0001
Spleen volume,cm^3^	224.4 ± 78.8	211.3 ± 71.7	0.4546

The values are expressed as mean ± standard deviation(SD)

Inflammation is a key feature of AAA onset as demonstrated by extensive medial and adventitial inflammatory cell infiltration into the vessel wall [5]. Previous studies have demonstrated a predominance of CD4^+^ T cells in AAA [6], which could derive from circulating monocytes mobilized from spleen [7]. Early monocyte increase in the blood could predicts the incidence of AAA formation in apolipoprotein E knockout (ApoE^−/−^) mice [8]. The spleen is important to the immune system as its ability to host extramedullary haematopoiesis [9, 10], indicating that the spleen may play an important role in inflammatory diseases and may reveal functional or even morphological changes. Among all the morphological changes, splenomegaly is a common radiologic and clinical sign, which may result from hematologic disorders, inflammatory or infectious diseases, etc. [11].

Currently, it has been proved that Angiotensin II (Ang II)–induced inflammatory reactions will cause splenic enlargement in Ang II/APOE model [12, 13]. Further study revealed that splenectomy before Ang II delivery would inhibit early monocyte subset mobilization into the blood and protect against AAA in experimental AAA model [8]. However, it remains unknown whether the AAA patients have similar features. Therefore, we hypothesized that there would be similar changes of the spleen size in AAA patients, which might be a predictor of the progression of AAAs. In this article, we investigated the potential mechanism in Ang II/APOE model and tested the associations between the size of the spleen and the indices of AAA in human based on a retrospective study of men.

## Methods

### Murine aneurysm model

Male C57BL/6 J and ApoE^−/−^mice (backcrossed 10 × into a C57BL/6 J background) were obtained from Department of Laboratory Animal Science (Peking University Health Science Centre, Beijing, China). All mice were bred as littermate controls, and housed in a pathogen-free barrier facility. Mice were fed a high-fat and high-cholesterol diet (1.25% cholesterol and 45% ghee) from 8 weeks of age. Male mice (8–10 weeks of age) were implanted with Alzet osmotic minipumps (Model 2004, Durect Corporation), filled either with saline vehicle or Ang II solutions (1000 ng/kg/min) up to 4 weeks [14]. The study cohort was composed of two groups: (1) Sham (*n* = 20); (2) Ang II (n = 20). For quantifying aneurysm incidence, an aneurysm was defined as a 50% or greater increase in the external width of the suprarenal aorta compared to aortas from saline-infused mice. This definition is consistent with a commonly used clinical standard to diagnose abdominal aortic aneurysm as an increase in aortic diameter of 50%, as described previously [15]. All studies were performed with the approval of the Institutional Animal Care and Use Committee of Chines Academy of Medical Sciences & Peking Union Medical College (CAMS&PUMC).

### Histological analysis

The mice were killed after 4 weeks of treatment. The mice were anesthetized with 2% pentobarbital solution (Catalog number: P3761, Sigma, USA) by intraperitoneal injection, and then sacrificed by bleeding through the carotid artery. For morphological analysis, aortas were perfused with normal saline and fixed with 10% PBS and formalin for 5 min. Whole aortas were harvested and the aortic tissue was removed from the ascending aorta to the iliac bifurcation, as well as the spleen. The tissue was laid out on a blue background, and an image of the aorta was recorded. After fixed for 24 h and embedded in paraffin, and cross-sections (5 μm) were prepared. Paraffin sections were stained with H&E and immunostaining,as described previously [14]. Antibodies to CD3 (Catalog number: BS-1521) were from Bioworld, and were used in a 1:100 dilution.

### Baseline clinic data collection

We retrospectively analyzed 432 male patients’ imaging (mean age 70 years, range from 44 to 88 years) from January 1, 2012 to December 31, 2014 in Shandong Medical Imaging Institution. All the patients underwent CTA. Exclusion criteria includes aneurysm surgery, patients’ age younger than 50 years or greater than 80 years, and other diseases leading to spleen size and/or shape changes such as tumors, cysts, infection, hematopoietic system diseases, the lymphatic system diseases, connective tissue disease, metabolic disease, autoimmune diseases and parasitic diseases, unexplained fever for a long time, etc. Besides, to rule out the influences of atherosclerosis on the control, 53 cases of male patients with abdominal aortic atherosclerosis were excluded. Eventually, 30 cases of male patients with AAAs (median age 69 years, range from 50 to 78 years) were included in the study. The median Dmax was 5.2 cm (ranged from 3 to 10.3 cm). A total number of 25 patients (median age 55 years, ranged from 50 to 68 years) with no anomalies in abdomen CT scan were selected as the control group. This was a retrospective study, and the institutional review board of Shandong Medical Imaging Research Institute, Shandong University, waived informed consent.

### CT scan method

All CT examinations were performed with a DSCT (Somatom Definition, Siemens Medical Solutions, Forchheim, Gemany). Imaging parameters were as follows: Detector collimation 2 × 32 × 0.6 mm; Slice collimation, 2 × 64 × 0.6 mm by means of a z flying focal spot, pitch 0.8; The tube current–time product was 380 mAs per rotation; For a patient of BMI < 30 kg/cm^2^, the tube potential was 100 kV, while for BMI > 30 kg/cm^2^, the tube potential was 120 kV. The injection protocol was biphasic: 1.2 ml/kg of non-ionic low-osmolar contrast material (Iohexol 370 mg I/ml) was injected, followed by 50 ml of saline flush. A bolus tracking technique was used for each acquisition: the region of interest (ROI) was drawn on the descending aorta, and image acquisition was started automatically after reaching a threshold of 100 HU in the ROI. Data sets were filtered with a medium-soft convolution kernel (B26f), with 1.5 mm thickness and 1 mm layer interval. Image was transited to pretreatment workstation (Syngo. Via VA 2.0, Siemens Healthcare, Germany.) to be reconstructed and diagnosed together by two experienced radiologists. AAA was defined as Dmax ≥3.0 cm or Dmax ≥1.5 × suprarenal aortic diameter [16]. For any disagreement between the two observers, consensus agreement was achieved.

### Analysis and quantification of spleen volume

We used Magnetic Resonance Imaging (MRI) to learn the morphological changes of the spleen in vivo after 4 weeks of treatment before killing the mice, as described previously [17]. Images were processed using the Bruker Paravision 4.0 program. When the mice were killed, the actual spleen volume was measured using liquid measure. For the measurement of spleen volume in human, all CT images were transferred to an external workstation (Syngo Via, Siemens Medical Solutions, Forchheim, Gemany) for volume measurement. We manually draw an outline of the spleen on each slice of the axial images. After calibration, the spleen volume is automatically calculated from the computer. Three doctors measured the spleen volumes and the average values were recorded. (Fig. 2a and b).

### Statistical analysis

Continuous variables are expressed as the means ± standard error (SEM) for the animal experiments and as means ± standard deviations (SD) for clinic research. Comparisons of parameters between two groups were made by *t* test. Comparisons of parameters among more than two groups were made by one-way analysis of variance (ANOVA), and comparisons of different parameters between each group were made by a post hoc analysis using a Bonferroni test. A χ^*2*^ test was applied to the comparisons of AAA incidence and survival rate. We tested for a correlation between spleen volume and the age or Dmax in all the patients and the AAA patients respectively, using the nonparametric Spearman correlation test. Statistical significance was evaluated with SPSS17.0 (SPSS Inc., Chicago, Ill). A value of *P* < 0.05 was considered to be statistically significant.

## Results

### Animal experiments

#### Ang II induces AAA concurrently with spleen volume enlargement in *ApoE*^−/−^ mice

**Fig. 1:**
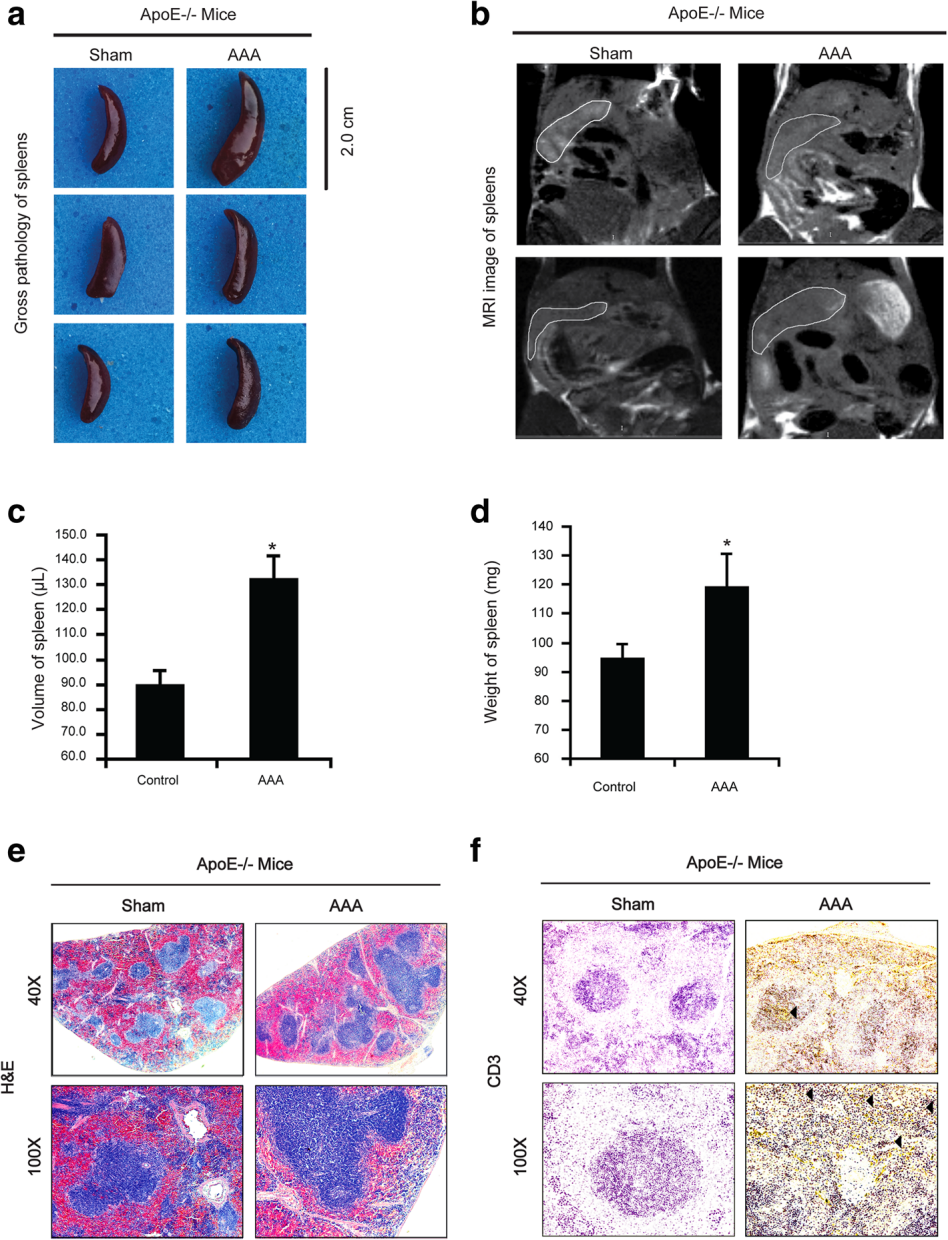
Gross, MRI and histopathological investigations of different experimental animal spleen. **a** Gross pathology of spleens of different experimental ApoE^−/−^ mice: Normal saline-treated Apoe^−/−^ mice show spleen with normal size and the Ang II-treated animals with AAAs show enlarged spleen (Right panel). **b** On T1wI the Ang II-treated animals with AAAs show enlarged spleens (Right panel) compared with the saline-treated ApoE^−/−^ mice (Left panel). **c** Larger volume in Ang II-treated animals with AAAs. Values are means ± SDs. *P < 0.05; **P < 0.01. **d** Higher spleen-to-body weight ratio in Ang II-treated animals with AAAs. Values are means ± SDs. *P < 0.05; **P < 0.01. **e** Left panel: Normal splenic follicles and periarteriolar lymphoid sheath (PALS) in saline-treated ApoE^−/−^ mice. Right panel: Hypertrophy of splenic follicles are increasingly seen in the medullary regions, large numbers of proliferative immature lymphocytes (arrow) in the lymphatic follicles and increase in the size of periarteriolar lymphoid sheath with resident T lymphocytes, in Ang II-treated animals with AAAs. **f** Immunostaining result reveals the large numbers of proliferative immature lymphocytes (arrow) in the lymphatic follicles in Ang II-treated animals with AAAs

Before harvest of the sample, MRI was performed showing the enlargement of spleen in mice with AAAs (Fig. 1b). During the harvest of aortic tissue on day 28 after Ang II infusion, we also found that most spleens in the Ang II group developed splenic enlargement, whereas few changed in the sham group (Fig. 1a). The volume of spleen and the spleen-to-body weight ratio were also analyzed by a manual measurement. Compared with the control group, both the spleen volume and the spleen-to-body weight ratio were increased accompanied with AAAs formation (Fig. 1c and d). Next, we conducted histopathological investigations on spleens of both group. Hypertrophies of splenic follicles were observed in mice with AAA (Fig. 1e, right panel) but not in the control (Fig. 1e, left panel). In addition, the number and size of the follicles in the medullary region were markedly increased in the spleens of mice with AAA as compared with those in the control. The proliferation of CD3^+^ T lymphocytes within the lymphatic follicles of mice with AAA appeared higher (Fig. 1e).

### Clinic cohort study

#### Demographic and clinical data

In 432 cases, 165 cases with basic lesions about spleen, 108 cases were abdominal aortic dissection, intramural aortic hematoma, postoperation, or abdominal aortic atherosclerosis, and 104 cases with age less than 50-year-old and greater than 80-year- old had been ruled out. At last, we enrolled 30 patients in the AAA group and 25 patients in the control. Statistical significant differences were found between the two groups with respect to age (*P* < 0.0001). Nonparametric Spearman correlation test results revealed that with the increase of age, spleen volume showed a shrinking trend among the whole population. However, among 108 patients between the ages of 50–80, the trend was nonsignificant (*r* = −0.1150; *P* = 0.2377) (Fig. 3b). Background charateristics of the patients are listed in Table 1.

### Quantitative analysis of spleen volume

We analyzed the spleen volume of all the patients as well as the Dmax using the method described above. There is not significant difference in spleen volume between the AAA group and the control group (211.3 ± 71.7 cm^3^ vs. 224.4 ± 78.8 cm^3^; *P* = 0.4546) (Table 1; Fig. 3a). As AAA with Dmax larger than 5.5 cm in man are defined as higher tendency for rupture, and considering the significant difference between the two groups with respect to age, we further divided the AAA group into two subgroups, which was defined as large AAA (Dmax > 5.5 cm) (*n* = 15) and small AAA (Dmax < 5.5 cm) (*n* = 15). We found significantly higher values in large AAA compared with the small AAA for spleen volume (230.6 ± 64.5 cm^3^ vs. 170.0 ± 32.8 cm^3^; *P* = 0.0030) (Fig. 3c).

### Correlation of spleen volume with Dmax

**Fig. 2:**
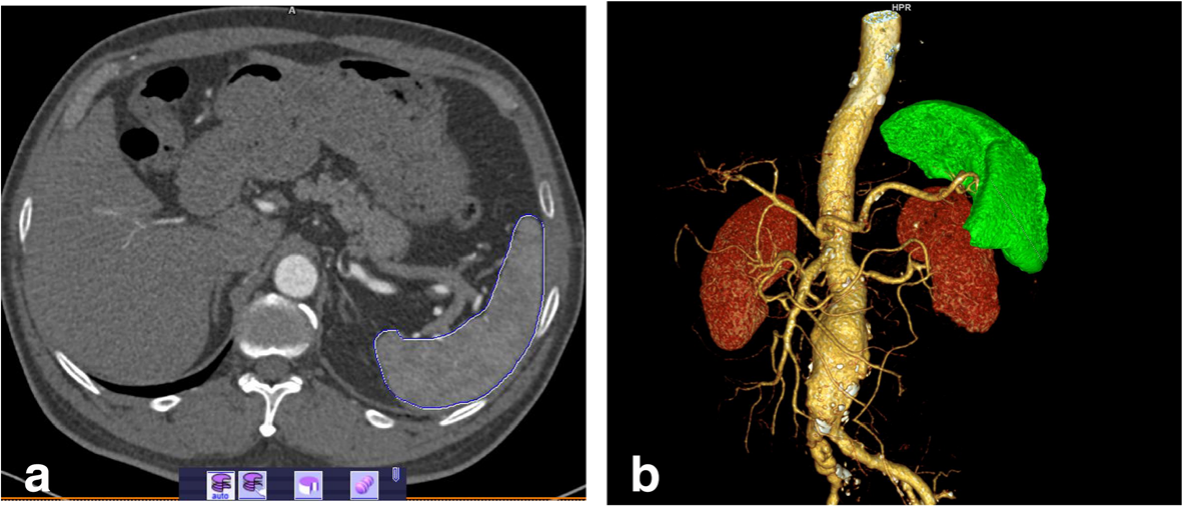
Representative CT images illustrating the outlining of the spleen. **a** Cross-sectional CT image shows measurement of spleen volume in an AAA patient indicated by a circle. **b** Measurement of spleen volume in three-dimensional model derived from the CT reconstruction of the AAA,indicated by segmentation of contrast extravasation (green)

**Fig. 3:**
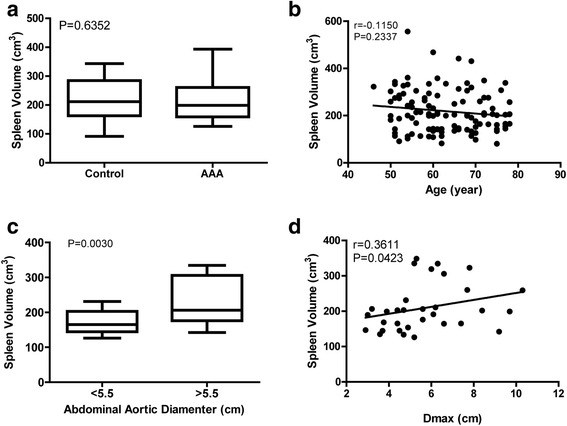
The changes in the spleen volume between the AAA group and the control group. **a** Boxplots of the spleen volume in the AAA group and the control group.There was no significant difference between the two groups (*P* = 0.4546). **b** Correlation of spleen volume with age among all the patients. The correlation between spleen volume with age was not significant (*P* = 0.2377). **c** Boxplots of the spleen volume in large AAA (Dmax > 5.5 cm) (*n* = 15) and small AAA (Dmax < 5.5 cm) (n = 15). There was significant difference between the two groups (*P* = 0.0167). **d** Correlation of spleen volume with Dmax among patients between 50 and 80 years old. There was a significant moderate positive correlation between spleen volume and Dmax (*r* = 0.3611; *P* = 0.0423)

For correlation of spleen volume and Dmax of AAAs, paired data sets were available from the 30 patients enrolled in the AAA group. We determined the correlation of Dmax of AAA with spleen volume and age. After performing regression analysis, we found a statistically significant positive linear correlation of spleen volume and Dmax of AAA (*r* = 0.3611;*P* = 0.0423) (Fig. 3d). In contrast, we did not find a linear correlation of spleen volume and age. Representative CTA images from an AAA-patient are shown in Fig. 2. Regression curves for the correlation of the spleen volume with Dmax of AAA as well as age are shown in Fig. 3.

## Discussion

Inflammation is a key feature of AAA onset, and CD4^+^ T cells infiltration in the vessel wall plays an important role. Spleen is an important secondary lymphoid organ, which is key to the function of T cells [18]. Besides, early monocyte mobilization from the splenic monocyte reservoir could potentially promote aneurysms rupture [9]. The findings indicate that during the progression of AAA, the spleen may play an important role. Unlike the bone marrow, the spleen is an organ that can rapidly expand in size, suggesting that the inflammation response may cause functional or even morphological changes of the spleen, which may have the potential for timely reflection of the inflammation of another organ.

Splenic enlargement may result from inflammatory/infectious diseases as well [19], and has been tested in several vascular diseases, such as myocardial infarction (MI) [20, 21]. Recently, in Ang II/APOE model, marked splenic enlargement was observed in all Ang II-treated mice, and the presence of lymphocytes attributed to the splenic enlargement [12]. Similarly, in elastase perfusion model of AAA, smoke exposure could significantly increased the percentage of CD3^+^ T cells in both of the abdominal aorta and the splenic tissue of the animals [22]. Consistent with those findings, our study also showed a significant increase of CD3^+^ T lymphocytes in the splenic tissue of mice with AAAs. However, to the best of our knowledge, whether the spleens reveal similar changes of size in human has not been elucidated.

To test the associations of AAA size with changes of the spleen volume, we retrospectively analyzed the difference of spleen volume between AAA group and the control group in human. Our results showed that with the increase of age, spleen volume showed a shrinking trend among the whole population (Fig. 3a) consistent with previous findings [23]. However, among 108 patients between the ages of 50–80, the trend was nonsignificant (*r* = −0.1150; *P* = 0.2337). Among those patients, we performed further analysis to find significantly higher values in large AAA (Dmax > 5.5 cm) compared with the small AAA (Dmax < 5.5 cm) for spleen volume (230.6 ± 64.5 cm^3^ vs. 170.0 ± 32.8 cm^3^; *P* = 0.0030) (Fig. 3c). Additionally, we found a statistically significant positive linear correlation of spleen volume and Dmax of AAA (*r* = 0.3611; *P* = 0.0423) (Fig. 3d). We hypothesized that in patients with large AAA and high risk of rupture, more inflammatory cells might be mobilized in the spleen and infiltrate the aorta to promote the local inflammation and the degradation of the aortic wall. And the lasting mobilization of the inflammatory cells causes the gradual increase of the spleen volume. However, the precise pathological mechanisms still remain elusive and need further investigations.

It has been well recognized that the main complication of untreated AAA is rupture, with more than 80% associated mortality [1]. The most widely used criteria to predict the risk of aneurysm rupture are the Dmax and gender, which are based on threshold values of 5.5 cm for men and 5.0 cm for women [3, 24]. It is clear from a population-based cohort study [25] and randomized trials [3] that the rupture risk increases with larger AAA diameter. However, the Dmax criterion should be revisited for several reasons. Recently with the technological advances in radiology, some morphological indices predictive of rupture risk have been identified [26, 27]. But more indicators are required to prove the sensitivity and specificity, especially for the prediction of asymptomatic AAA. Because inflammation is the key pathological feature of AAA and plays different roles in different stage of AAA, it may be the soil for digging out the predictors of AAA prognosis. By now, several studies have proved that some cytokines and chemokines produced during the inflammatory response could show the stage of AAA. Therefore, we hypothesized that the changes of the spleen size may be a predictor of the prognosis of AAA. Our clinical findings are consistent with the pathological features of murine AAA model, suggesting that the change of spleen volume could be an indicator for estimating the rupture risk of AAA. The explanation for the enlargement has been proved in animal experiments, but further study is required to testify it in human.

However, there are some limitations in this study. First, our study is retrospective cross-sectional design. According to the report in the literature, normal spleen volume is affected by some factors including age, gender, height, weight, etc. [23]. To reduce the influence, we decided to select the male patients between the age of 50–80, among which there was nonsignificant correlation of the age and spleen volume. But limited to the study design, we could not collect the data of height, weight to adjust the spleen volume using previous method [23]. But, as Geraghty et al. [23] have revealed that the correction for height and weight usually resulted in a small change in organ volume, our outcomes may be representative for the actual results. However, corrections for patients who are quite thin or heavy will show a larger change, which will affect the power of our conclusions. Besides, the expansion rate of the AAA could not be available for analysis. Therefore, further researches are required. Second, the sample size was relatively small, making it less powerful to analyze the difference between the AAA and the control group because of the variance of the spleen volume. Third, the patients enrolled in this study were enrolled in the single center. Therefore, some bias could be present between the groups.

## Conclusions

In conclusion, our study demonstrates that the changes of spleen size in patients with AAA revealed the similar pattern with the murine AAA model. Our data further demonstrated a positive correlation between spleen volume and the Dmax in male AAA patients. Based on these data, we hypothesize that the spleen expansion possibly contributing to or as a consequence of the progression of AAA, and may be a potential indicator of the risk of rupture. To our knowledge, this is the first time to report clinic data showing the association of changes in spleen volume and the Dmax of AAA. Because there were some limitations in our study, further studies are warranted to address these hypotheses and better delineate the potential significance of splenic enlargement to the prognosis of AAAs.

## Abbreviations

AAA: abdominal aortic aneurysm
Ang II: angiotensin II
ANOVA: one-way analysis of variance
ApoE^−/−^: apolipoprotein E knockout
CTA: computed tomography angiography
Dmax: the maximum abdominal aortic diameter
MI: myocardial infarction
MRI: magnetic resonance imaging
ROI: the region of interest
SD: means ± standard deviations
SEM: means ± standard error
